# Complete Genome Sequence of the Naegleria fowleri (Strain LEE) Closed Circular Extrachromosomal Ribosomal DNA Element

**DOI:** 10.1128/MRA.01055-20

**Published:** 2020-12-03

**Authors:** John C. Mullican, Kristen M. Drescher, Nora M. Chapman, Steven Tracy

**Affiliations:** a Department of Pathology and Microbiology, University of Nebraska Medical Center, Omaha, Nebraska, USA; b Department of Medical Microbiology and Immunology, Creighton University Medical School, Omaha, Nebraska, USA; Indiana University, Bloomington

## Abstract

The circular extrachromosomal ribosomal DNA (rDNA) element of Naegleria fowleri strain LEE was molecularly cloned and fully sequenced. The element comprises 15,786 bp and contains a single copy of the organism’s rDNA cistron. The nonribosomal sequence contains five potential open reading frames, two large direct repeat sequences, and numerous smaller repeated-sequence regions.

## Abstract (precis)

The circular extrachromosomal ribosomal DNA (rDNA) element of Naegleria fowleri strain LEE was molecularly cloned and fully sequenced. The element comprises 15,786 bp and encodes a single copy of the organism’s rDNA cistron. The nonribosomal sequence contains five potential open reading frames, two large direct repeat sequences, and numerous smaller repeated-sequence regions.

## ANNOUNCEMENT

Naegleria fowleri (Heterolobosea, Schizopyrenida, Vahlkampfidae, Naegleria) causes primary amebic meningoencephalitis, a usually fatal brain infection (1–3). *Naegleria* species contain a single ribosomal DNA (rDNA) cistron (5.8S, 18S, and 28S rRNA genes) on closed circular extrachromosomal rDNA elements (CEREs); there are approximately 4,000 copies per cell (4–7). Genome sequencing of three *Naegleria* species (8–11) confirmed that there are no rDNA genes in the nuclear genome. Here, we report the complete sequence of the N. fowleri (strain LEE) CERE. While the total genome of N. fowleri (strain LEE) has also been sequenced (10), this report demarcates for the first time the complete rRNA coding sequences of N. fowleri and identifies five putative open reading frames (ORFs) and repeated sequences in the nonribosomal sequence (NRS).

N. fowleri LEE strain amebae, kindly provided by David John (Oklahoma State University, Tulsa, OK, USA), were propagated axenically at 37°C in stationary tissue culture flasks in Nelson’s medium (12) supplemented with 4% (by volume) calf serum. Total DNA was extracted from log-phase N. fowleri trophozoites, collected centrifugally, and suspended and lysed in a highly chaotropic lysis buffer (8 M NaSCN, 50 mM Tris-HCl [pH 7.5], 50 mM EDTA [pH 8.0], 5 mM EGTA, and 142 mM β-mercaptoethanol) as described previously (7). Following electrophoresis in 0.8% agarose gels in 0.2× Tris-acetate-EDTA (TAE) buffer and staining with ethidium bromide, the 16-kbp supercoiled CERE, migrating in front of the genomic DNA band, was visualized by UV illumination, excised, and purified free from agarose. The isolated CERE was linearized with HindIII and ligated into the pGEM7Zf(+) vector (Promega Corp.).

The complete sequence was assembled from clones, subclones, and purified uncloned DNA. Illumina next-generation sequencing (NGS) using the NextSeq system was outsourced to Applied Biological Materials (Richmond, BC, Canada), and Sanger sequencing (13) using AB 3500xL genetics analyzers was outsourced to Eurofins Genomics (Louisville, KY). DNA for Illumina sequencing was subjected to tagmentation and adaptor-mediated amplification using the Nextera XT DNA library preparation kit (Illumina), following the vendor’s protocol. The final size selection to remove adapter dimers was performed using magnetic beads for DNA purification (Applied Biological Materials). Following library preparation, the samples were run on an Agilent Bioanalyzer using the DNA 1000 kit (Agilent) to confirm the size distribution (a bell curve distribution with a peak between 400 and 600 bp) and to ensure that all adapter dimers had been removed (no peak visible in the 120- to 150-bp range). Samples were quantified by quantitative PCR using the KAPA library quantification kit for the Illumina platform prior to pooling and sequencing on a NextSeq 500 instrument (Illumina). NGS produced 554,274 paired-end reads of 72-bp average length, totaling 81 Mb; Sanger sequencing produced 118 reads totaling 91.7 kb, representing ∼6× coverage in both directions.

The first 25,000 pairs of Illumina reads, representing optimal ∼200× coverage of the target plasmid, were assembled using SPAdes version 3.11.0 (removing reads of <33 bases and reads with a Phred score of <20) (14) as implemented in MacVector version 16.0.10 (MacVector, Inc., Cary, NC), resulting in a single linear contig of 17,938 bp. The assembly software utilized default parameters unless otherwise noted. Attempted validation of the consensus using the Align to Reference tool in MacVector with the Illumina reads indicated multiple regions in which the assembly was likely inaccurate due to the presence of multiple direct and inverted repeats. Within MacVector, Sanger reads, which had been base called using Phred, were used in conjunction with the Align to Reference function and the Phrap assembler (version 1.090518) to resolve the repeats and to generate a final circular plasmid sequence of 15,786 bp after removal of the cloning vector sequences.

The 18S, 5.8S, and 28S rRNA gene sequences were identified by search and alignment of Rfam families (15) using the Rfam 14.2 database. ORFs of ≥100 codons on both strands were identified (16). Repeated sequences were identified by BLAST (17) analysis of the entire sequence versus itself using MegaBLAST settings.

**FIG 1:**
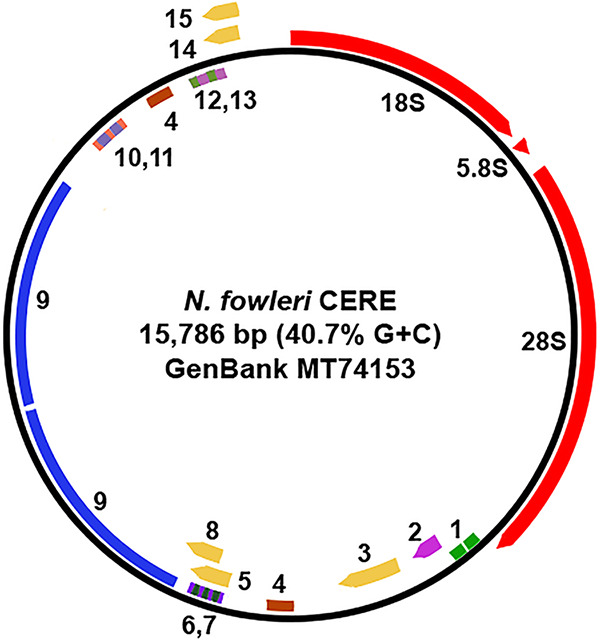
Map of the CERE from N. fowleri strain LEE. Features of the CERE are as listed for GenBank accession number MT741533. 18S, 5.8S, and 28S refer to the rRNA gene coding sequences. Repetitive DNAs are indicated as follows: 1, 168-bp tandem direct repeat; 4, 248-bp direct repeat; 6 and 7, 47- and 56-bp direct repeats, respectively; 9, 2,135-bp direct repeat; 10 and 11, 70- and 130-bp direct repeats, respectively; 12 and 13, 35- and 85-bp direct repeats, respectively. 2 indicates the group 1 intron. ORFs on the sense (or rRNA-encoding) strand are labeled 3, 5, and 8, and ORFs on the antisense strand are labeled 14 and 15.

An overview of the CERE features, including rRNA genes, ORFs, and repeat sequences, is depicted in Fig. 1. The CERE comprises 15,786 bp (overall GC content, 40.7%). The rDNA region is 5,863 bp (GC content, 46.5%), including two internal transcribed spacers, while the NRS is 9,923 bp (GC content, 37.6%). The NRS contains 10 repetitive DNA sequence families (expect values from 0.0 to 2e−9), accounting for 6,488 bp (65.3%) of the NRS and 41.1% of the total CERE DNA. Repeats range in size from 35 bp to 2,135 bp.

The NRS contains five putative ORFs, three on the rRNA-encoding strand and two on the opposite strand. Interestingly, only one of these (at nucleotides 6809 to 7435) is conserved (84% for DNA and 78% for the predicted amino acid sequence) in the CERE NRS of Naegleria lovaniensis, the species most closely related to N. fowleri (9), suggesting that this ORF alone may potentially encode a protein.

### Data availability.

The complete assembled sequence has been deposited in GenBank under the accession number MT741533. The version described in this paper represents the first version, MT741533.1. The publicly available raw sequence data for Sanger and Illumina NGS reads have been deposited in the Sequence Read Archive (SRA) under the accession numbers SRX8926575 and SRX8926574, respectively, and collectively under BioProject accession number PRJNA656555.
